# Histogenetic Radial Models as Aids to Understanding Complex Brain Structures: The Amygdalar Radial Model as a Recent Example

**DOI:** 10.3389/fnana.2020.590011

**Published:** 2020-11-10

**Authors:** Elena Garcia-Calero, Luis Puelles

**Affiliations:** Department of Human Anatomy, School of Medicine and IMIB-Arrixaca Institute, University of Murcia, Murcia, Spain

**Keywords:** morphological models, neurogenesis, radial migration, subpallium, pretectum, thalamus, prethalamus, hypothalamus

## Abstract

The radial dimension expands during central nervous system development after the proliferative neuroepithelium is molecularly patterned. The process is associated with neurogenesis, radial glia scaffolding, and migration of immature neurons into the developing mantle stratum. *Radial histogenetic units*, defined as a delimited neural polyclone whose cells share the same molecular profile, are molded during these processes, and usually become roughly stratified into periventricular, intermediate, and superficial (subpial) strata wherein neuronal cell types may differ and be distributed in various patterns. Cell-cell adhesion or repulsion phenomena together with interaction with local intercellular matrix cues regulate the acquisition of nuclear, reticular, or layer histogenetic forms in such strata. Finally, the progressive addition of inputs and outputs soon follows the purely neurogenetic and radial migratory phase. Frequently there is heterochrony in the radial development of adjacent histogenetic units, apart of peculiarities in differentiation due to non-shared aspects of the respective molecular profiles. Tangential migrations may add complexity to radial unit cytoarchitecture and function. The study of the contributions of such genetically controlled radial histogenetic units to the emerging complex neural structure is a key instrument to understand central nervous system morphology and function. One recent example in this scenario is the recently proposed *radial model of the mouse pallial amygdala*. This is theoretically valid generally in mammals (Garcia-Calero et al., 2020), and subdivides the nuclear complex of the pallial amygdala into five main radial units. The approach applies a novel *ad hoc* amygdalar section plane, given the observed obliquity of the amygdalar radial glial framework. The general relevance of radial unit studies for clarifying structural analysis of all complex brain regions such as the pallial amygdala is discussed, with additional examples.

## Introduction

**Figure 1:**
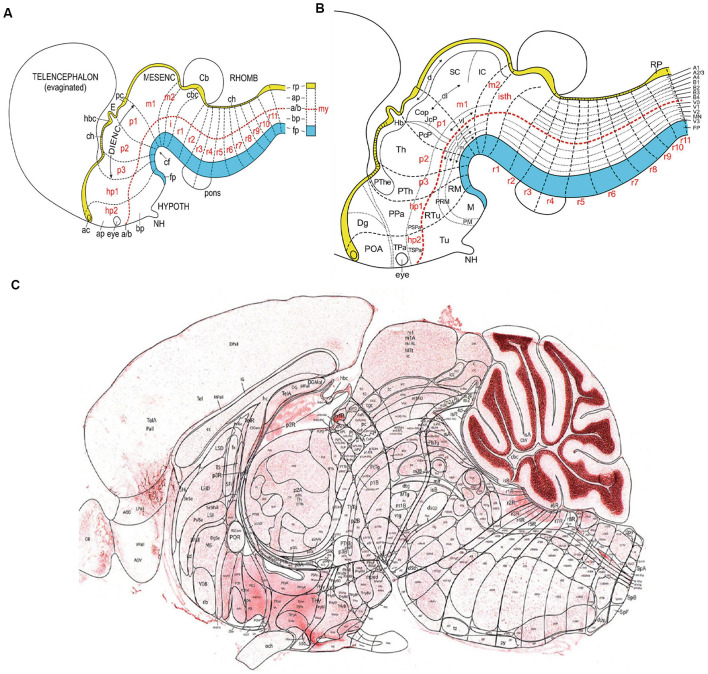
**(A)** Schema illustrating the set of primary dorsoventral or axial longitudinal zones [*floor plate* in blue (fp); *roof plate* in yellow (rp); the *basal* and *alar plates* (bp, ap) are separated by the red dashed alar-basal longitudinal border, a/(b)], intersected orthogonally by anteroposterior transverse units (hypothalamic prosomeres hp1–2, diencephalic prosomeres p1–p3, midbrain prosomeres m1–m2, and hindbrain rhombomeres isth/r0-r11; a symbolic spinal neuromere is added in **A**; all neuromeres are identified in red letters). This check-board pattern represents the primary regional subdivisions of the brain wall; they are partially deformed by the bends in the neural tube axial dimension (cf; cephalic flexure). Note rostral separate endings of the floor, alar-basal boundary, and roof domains at the acroterminal end of the neural tube. Taken from Nieuwenhuys and Puelles (2016), plate 93. **(B)** Schema similar to panel **(A)** showing a more advanced stage, in which some primary regions have started to regionalize (subdivide secondarily), either anteroposteriorly (e.g., see alar pretectum or p1), or dorsoventrally (e.g., brainstem and forebrain alar and basal plates). These subdivisions lead to smaller molecularly defined progenitor regions, which may eventually be definitive. Taken from Nieuwenhuys and Puelles (2016), plate 95. **(C)** Essentially a similar schema, now of definitive areal partitions or fundamental morphological units (FMUs) drawn upon a real parasagittal adult mouse brain section (from P56 reference atlas in the Allen Developing Mouse Brain Atlas; original drawing of LP). Unequal growth over pre- and postnatal development has caused some neuromeric units to result compressed (mostly at the floor area at the cephalic flexure), variously bent, or deformed in directions not seen in this section (e.g., telencephalon, eyes, cerebellum, not subdivided in this schema). Nevertheless, the primary units in panel **(C)** correspond exactly to those in **(A,B)**, showing now their definitive regionalization into a multitude of FMUs, where specific neuronal types are produced. It can be seen that most anatomic regions of the brain are composed of several FMUs (e.g., the hypothalamus). Taken from Nieuwenhuys and Puelles (2016), plate 92. Abbreviations: **(A)** ac, anterior commissure; CB, cerebellum; ch, chorioidal tela; E, epiphysis; IC, inferior colliculus; my, myelomere (spinal cord segment); NH, neurohypophysis. **(B)** The CoP, commissural pretectum; Dg, diagonal subpallial area; Hb, habenula; JcP, juxtacommissural pretectum; M, mamillary area; NH, neurohypophysis; PcP, precommissural pretectum; PM, perimamillary area; PRM, peri-retromamillary area; POA, preoptic area; PPa, peduncular paraventricular area; PSPa, peduncular subparaventricular area; PTh, prethalamus; PThE, prethalamic eminence; RM, retromamillary area; RTu, retrotuberal area; SC, superior colliculus; Th, thalamus; TPa, terminal paraventricular area; TSPa, terminal subparaventricular area; Tu, tuberal area. **(C)** Consult the Allen P56 Reference Atlas associated to the Developing Mouse Brain Atlas. This Reference Atlas was designed and executed by LP.

In terms of causal morphogenesis, the pallial amygdala is a typically badly understood part of the mammalian telencephalon. It consists of a bundle of heterogeneous nuclear cell masses (the proverbial “bag of potatoes”), whose reasons to be where they are remain completely unexplored. It can be safely said that current literature ignores the number and type of radial histogenetic units that shape up the pallial amygdala of mammals and non-mammals. Recently we elaborated a radial model of the mouse pallial amygdala, aiming to start to unravel the conceptual difficulties implied in understanding causally such a complex nuclear structure (Garcia-Calero et al., 2020). In both classic and modern descriptions of the rodent pallial amygdala (e.g., Johnston, 1923; Krettek and Price, 1978; De Olmos et al., 1985, 2004; Alheid et al., 1995; Paxinos and Franklin, 2007, 2013), individual nuclei are distinguished and named by their apparent relative topographic position in a coronal section series. This approach wrongly assumes that a method that is largely useful for the telencephalic cortex remains useful for the amygdalar region. However, coronal sections imply an arbitrary axis of reference (usually that of the brain atlas used), which is generally based on the now outdated columnar tradition assuming that the forebrain axis ends in the telencephalon; see criticism in Puelles and Rubenstein (2015). The actual axis used is usually that of a stereotaxic instrument, given a fixed position of the animal head. This approach leaves out of consideration the peculiar relationships of the amygdalar nuclear complex with the corresponding ventricular surface (found *behind* the group of amygdalar nuclei) and the respective pial surface (*ventrocaudal* to the complex). Such a topographic approach on coronal sections was a standard during the classic period of columnar neuroanatomy, and was unwittingly applied likewise to other complex brain regions (e.g., subpallium, thalamus, hypothalamus, bed nucleus of stria terminalis, parabrachial hindbrain complex). As a result, causal morphologic understanding of these brain regions still escapes us at least in part, insofar as the columnar rather than the neuromeric paradigm (Figure 1) was traditionally applied to them, with the consequent deficits (i.e., lack of theoretical concepts able to explain the complexity of boundaries and differential local histogenetic and morphogenetic phenomena in the brain).

Conversely, the ontogenetic subdivisional concepts enabled by the neuromeric paradigm can be applied not only to neuromeric derivatives but also to particular non-neuromeric parts of the brain such as the important structural complexes found within the evaginated telencephalic vesicle, which is only indirectly related to a prosomere (Puelles et al., 2012; Puelles and Rubenstein, 2015). The telencephalon derived in the earliest vertebrates from a hyperdorsal radial unit of the alar hypothalamus, within the hypothalamo-telencephalic prosomere 1 (Figure 1; Puelles et al., 2012). Its early evagination and subsequent varied expansion in vertebrates allowed the emergence of several peculiarly structured neural regions in addition to the cortical pallium, of which the subpallium, the septum, and the amygdala are examples. Each of these complex domains is susceptible to a study in terms of distinct radial units, since they contain different sectors, both structurally and molecularly (e.g., subpallium model in Puelles et al., 2013).

The conceptual approach at the basis of the mouse *amygdalar radial model* (Garcia-Calero et al., 2020) would be useful for examining other brain regions. Such an approach was applied, perhaps somewhat unwittingly (because developmental notions were not foremost in the relevant adult neuroanatomic scenario), when Heimer and colleagues started examining the radial relationships of subpallial masses with the respective ventricular and pial surfaces (Heimer et al., 1982, 1991, 1995, 1997, 1999; Heimer and Alheid, 1991; reviewed in Heimer et al., 2008). These authors came up with the novel notion that the mammalian caudate and putamen subpallial nuclei relate radially to each other as periventricular and intermediate strata of a particular radial unit (the striatum), whose superficial component was detected in the ventral striatum and associated olfactory tuberculum. We later proposed a consistent application of the same approach to all subpallial masses in our model of the mammalian subpallium (Puelles et al., 2013, 2016c). It may be of interest that, shortly before his death in 2007, Heimer contacted L. Puelles to express interest in the application of the then-emerging *molecular developmental tool-kit* to his ideas of subpallium and extended amygdala. Publications were interchanged, and this impulse led (jointly with other circumstances) to the Flames et al. (2007) report on molecularly defined microzones in the mouse subpallium. The Puelles et al.’s (2013) subpallium radial unit model, ulteriorly used by Silberberg et al. (2016) in the analysis of relevant cis-regulatory enhancer analysis, clarified the general existence of periventricular, intermediate, and superficial strata as a characteristic of four distinct subpallial radial units, identified as the striatum, pallidum, diagonal area and preoptic area, which extend from the septum into the amygdala. Heimer’s *de facto* radial unit conception of the subpallium, even if defined strictly on adult structure, opened the door to a “New Anatomy,” as was pointed out posthumously in Heimer et al. (2008). This new approach illuminated the clinical application of numerous accruing scientific data, as well as subsequent steps in the causal analysis (Flames et al., 2007; Puelles et al., 2013, 2016c; Silberberg et al., 2016). The Puelles lab also contributed to the radial unit analysis of other parts of the chick or mouse brain. These involved the pretectum (Ferran et al., 2007, 2008, 2009), the thalamus (Redies et al., 2000; Martínez-de-la-Torre et al., 2002), the prethalamus (Puelles et al., 2020), the hypothalamus (Morales-Delgado et al., 2011; Puelles et al., 2012; Díaz et al., 2015; Ferran et al., 2015; Puelles and Rubenstein, 2015), and the isthmus (Puelles and Martinez-de-la-Torre, 1987; Watson et al., 2017).

The radial structure approach applied to either embryonic or adult material should unravel the histogenetic complexity of all complex neural regions, bearing implicitly on our potential future understanding of their specific patterning and histogenetic mechanisms. The capacity of this approach to illuminate as well as comparative studies on the evolution of such complex brain regions is likewise evident, due to the high evolutionary conservation of basic structural patterns.

In the following sections the radial histogenetic unit concept, together with details of the recent radial model of the pallial amygdala will be covered in the appropriate contexts, delving on the approach and its consequences as an example of recent progress in this field of the New Neuromorphology (Nieuwenhuys and Puelles, 2016; Nieuwenhuys, 2017).

## Radial Histogenetic Unit Concept

Areal units of neuroepithelial development were first conceived as “migration areas” by Palmgren (1921); Holmgren (1922, 1925); Rendahl (1924); Bergquist (1952), and Bergquist and Källén (1953a,b, 1954, 1955) within a variety of neuromeric studies. Puelles and Bendala (1978) studied migration and cell type differentiation in the developing chick optic tectum conceived as a large gradiental radial unit producing a rich set of neuronal types. This approach was employed as well by Vaage (1973) and Puelles and Martinez-de-la-Torre (1987) in studies of the chicken isthmic nuclei. Whole-mount analysis of the forebrain neurogenetic pattern based on the mapping of acetylcholinesterase-labeled young neurons in the chick led to the concept of “radial histogenetic areas,” which was influenced by the Nordic authors cited above (Puelles et al., 1987). Subsequently, several fate-mapping studies also *de facto* performed rough versions of radial histogenetic analysis in the chick hindbrain and diencephalon by analysis of whole neuromeres (Marín and Puelles, 1995; Cambronero and Puelles, 2000; García-López et al., 2004).

All these studies stood out among other ontogenetic studies by using a segmental (neuromeric) paradigm (Figure 1). That is, they all postulated a transversal (anteroposterior) neuromeric division of the neural tube, subdivided dorsoventrally in a first step by the four longitudinal zones (floor, basal, alar, and roof plates) defined previously by His (1893a,b, 1895, 1904). Irrespective of advancing axial bending of the neural tube, the topologically orthogonal intersection of transversal and longitudinal boundaries defined a checkerboard pattern of neuroepithelial areas, which can be studied as relatively independent units concerning specific variables (e.g., maxima of mitotic divisions and radial cell migration at their centers; Bergquist and Källén, 1954).

This segmental conception was for a century a heterodox paradigm, though it emphasized the interest of *differential cellular events* happening *in the brain wall* for understanding boundary formation. The opposed and dominant columnar school instead attended to *ventricular sulci* (Herrick, 1910, 1948), whose positions are much more difficult to account for molecularly than cellular differential details. With time, attention to sulci did not favor causal explanation (e.g., Swanson, 2012). The neuromeric approach led historically to *histogenetic* radial analysis within orthogonally delimited areal progenitor domains, the subject of the present essay. In this aspect, it contrasts with the historically preponderant orthodox columnar paradigm (Herrick, 1910; Kuhlenbeck, 1927, 1973). The latter emphasized during 100 years study of longitudinal sulcal boundaries (accidents of the ventricular relief) stretching theoretically across the whole brain (and only partially in agreement with the longitudinal zones of His, loc.cit.). The sulci were held to separate masses of neurons different in *function* (without an explicit theory of the biological grounds of such an association). The columnar paradigm unfortunately assumed a uniform structure, neuronal composition, and developmental pattern of large expanses of the neural wall thought to serve the same hypothetic function. This led consequently to “wavelike” interpretations of observed neurogenetic or vascularization patterns, thought to “spread” out of an early seed point in the medulla oblongata in rostral and caudal directions, without interposed boundaries or areal distinctions. No theoretic set of ideas developed within this paradigm to assist the analysis of more precise differential histogenesis. For instance, columnar authors assumed that the entire hindbrain somatosensory column was homogeneous since all rostrocaudal parts (actually nine neuromeric subunits) shared the same trigeminal function; the hypothalamus also was held to be structurally homogeneous, seen simplistically as a ventral (basal) diencephalic columnar unit. Present-day authors still following columnar tradition, such as Swanson (2012), treat the hypothalamic nuclear complexities they nowadays explore in great detail as neural phenomena devoid of conceivable causal morphogenetic explanation. This explanatory impasse has become particularly evident with the arrival of molecular delimitation methods, which have turned the rounds in favor of the segmental/neuromeric paradigm, its natural forebrain axis, and areal histogenetic units, with its promise of meaningful causal analysis.

Recently, Nieuwenhuys and Puelles (2016) coined in their book “Towards a New Neuromorphology,” also based on the segmental brain paradigm, the term *fundamental morphological units* (FMUs). This term describes as the fundament of adult sectorial morphology of the brain a still incompletely mapped set of radial histogenetic domains of the neural wall, which encompass the full ventriculo-pial radial dimension (e.g., Figure 1C). These domains derive directly *via* various phases of molecular regionalization from the larger longitudinally and transversally delimited early basal and alar progenitor domains contemplated previously in segmental/neuromeric models (Figures 1A,B). Essentially, each basal or alar domain of a neuromere (exceptionally, also its floor or roof plate) results subdivided secondarily by further regionalization into a specific set of molecularly differentiated *microzones*, which represent the definitive FMUs. The best-known examples are the five basal and six alar microzones of the spinal cord segments (Figure 1B, vertical list of areas at extreme right end of the image; reviewed in Puelles, 2013). It is not yet clear, due to deficient information on various parts of the brain, whether the FMUs show a common subdivision pattern throughout the spinal, hindbrain, and forebrain tagmata (Plate 95 in Nieuwenhuys and Puelles, 2016, reproduced here as Figure 1B). Large vesicular outpouchings originated from given neuromeric loci (such as the telencephalic vesicles) entail large expanses in surface growth, which may show exceptional degrees of singular regionalization and a realization into potentially evolutionarily variant numbers of FMUs in different species (e.g., number of cortical areas definable in the telencephalic pallium of diverse mammalian species; Kaas, 2017). Rakic (1988) proposed a “radial areal unit hypothesis” for cerebral cortical development (review in Rakic, 2007). This hypothesis stands upon pioneering experiments of cell proliferation in the cerebral cortex, the description of ventricular and subventricular progenitor zones in this region, and the study of neuronal migration into the cortical plate, guided by radial glia processes (Rakic and Sidman, 1968; Rakic, 1968, 1988, 2007). The distinct radial columns that subdivide the adult cerebral cortex form from clonally related cells, generated at the same place in the ventricular zone and with similar neurogenetic timing (Rakic, 1988, 2007). However, the case of the telencephalic cortex is not a general phenomenon, since, in contrast, the neural retina within the eye vesicles also grows enormously in surface, but retains the properties of a single repetitive radial unit or FMU (i.e., is not subdivided into distinct areas). The cerebellum is also a domain that expands significantly, but it retains a strictly uniform cellular structure (Figure 1C), aside from some regional rostrocaudal peculiarities (vermis, hemispheres, and auricle).

Progressive regionalization is assumed to obey continued patterning of the primary large neuroepithelial units in response to positional information triggering differential genomic readout. The positional information is provided gradientally by a variety of known, plus other as yet unknown, secondary organizers and their secreted morphogens (case of the isthmic organizer), or by cell-cell interactions between neighboring neuroepithelial areas (case of spinal cord dorsoventral patterning). Once completed, regionalization results in discrete areal loci of the neuroepithelium that display differential molecular properties *uniformly* (i.e., patches of matrix cells that share a unique specific molecular identity in terms of expressed/repressed transcription factors; this may include some factors shared with neighboring units). Implicitly, these newly parcellated areas have acquired *differential fates* thanks to their distinct molecular profiles.

**Figure 2:**
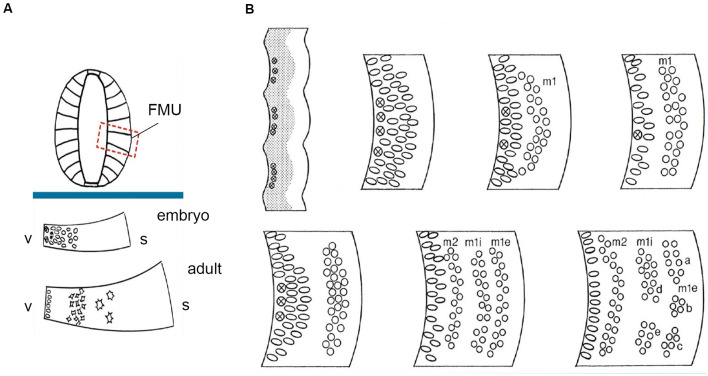
FMU concept: basic radial structure (extending from the ventricular surface –v- to the pial surface-s) and diversified formation of the neuron-rich mantle layer during development according to the unique combination of genetic background emerged within each one of them, allowing selective production of neuronal types. **(A)** above the blue trace, dorsoventrally delimited secondary radial units are shown in a symbolic neural tube transversal section (FMUs largely subdivide the basal and alar plates of the lateral neural tube wall; compare Figure 1B). Under the blue trace, we see two schemata of the same FMU observed in an embryo and an adult. These schemata illustrate first the embryonic production of undifferentiated neurons, and later the stratification and differentiation of these cells along the radial dimension of the mantle layer. Taken from Nieuwenhuys and Puelles (2016), plate 10. **(B)** At the upper left corner, a stretch of the neuroepithelial wall appears divided into separately bulging neuromeric FMUs. The ventricular zone is gray and contains mitotic cells (most numerous at the center of each FMU; the limits are hardly proliferative). The immature mantle zone (white) is still thin before neurogenesis begins. The other images in panel **(B)** show a schematic temporal neurogenetic sequence, as observed at a particular FMU (a different FMU might show a different pattern): there occurs initially formation of a first neuronal migration layer (m1), composed of postmitotic young neurons, which separates from the proliferating ventricular zone, before a second migration layer emerges (m2), which stabilizes deep to m1. In the meantime, the m1 layer population has subdivided radially into inner and external sublayers (m1i, m1e), whose “pronuclear strata” may later segregate and reaggregate into distinct nuclei. Each FMU has its pattern, though some adjacent FMUs may show similar characteristics. This histogenesis is the result of waves of mitotic activity in the progenitor zone, as well as of inherited differential adhesivity properties that mediate characteristic cell-cell aggregation or dispersion. Taken from Nieuwenhuys and Puelles (2016), plate 83.

**Figure 3:**
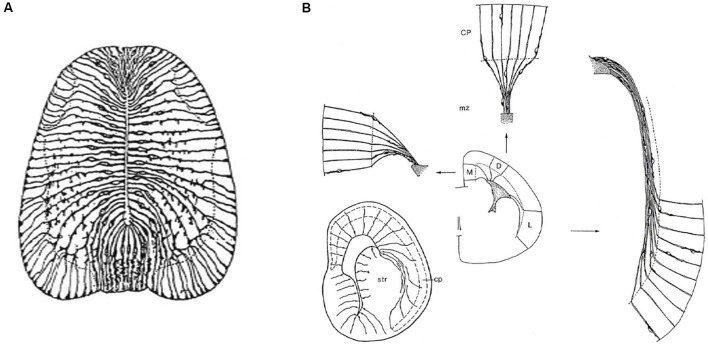
Packets or fields of radial glia constitute the skeleton of each FMU. By stretching and other adaptative deformations over development, they maintain the spatial correlation of the ventricular and pial surfaces of the FMUs. Brain wall morphogenesis thus leads to more or less deformed radial glial domains, which establish which topologically deep cells correspond to particular groups of superficial cells, thus identifying diverse units of the brain wall and indicating their true radial dimension. **(A)** Drawing by Cajal illustrating the disposition of radial glia at the floor, basal, alar, and roof plates of the spinal cord of a chick embryo (note the cell bodies aggregate at the ventricular zone; collateral filopodia decorate glial processes where they coexist with neurons in the mantle layer; note also some glial processes subdivide and become smoother as they relate at the spinal white matter with masses of fibers stretching their arrival to the brain surface). From Nieuwenhuys and Puelles (2016), plate 65. **(B)** Examples of medial, dorsal, and lateral radially deformed FMUs as detected by their glial skeletons at a given stage of telencephalic development. The radial glial processes, corresponding to lines guiding neuronal radial migration into the cortical plate (migratory zone or mz), indicate implicitly boundaries between distinct FMUs (e.g., pallio-subpallial boundary) and other sorts of morphogenetic deformations due to unequal *neuronal accumulation* at different parts of the walls of a telencephalic vesicle (e.g., at the striatum–str- compared with the cerebral plate–cp. Each sector (D, M, L) of the prospective cortex also shows differential glial deformation patterns, though the initial radial topology of each FMU remains unchanged. Taken from Nieuwenhuys and Puelles (2016), plate 82.

**Figure 4:**
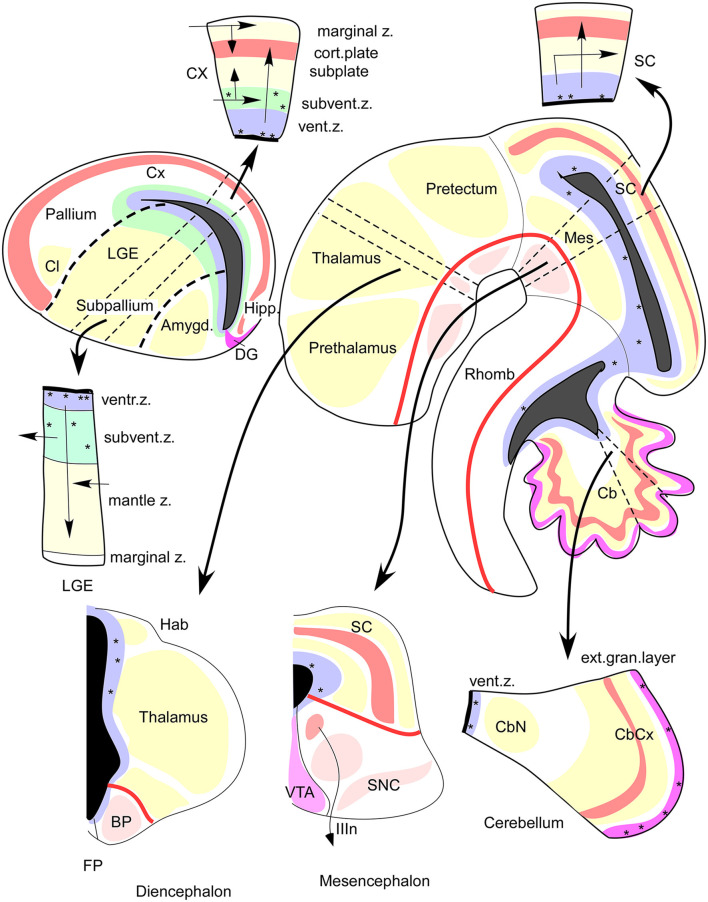
FMUs developing in different alar or basal parts of the neural wall, or in diverse anteroposterior brain regions, show site-specific proliferative, neurogenetic, and histogenetic properties due to their unique molecular profiles. This leads to variable degrees of stratification or of differentiation of nuclear vs. reticular neuronal formations in the mantle layer, always guided by the local (more or less deformed) radial dimension provided by the radial glia (not shown). The main central schema shows a lateral sagittal section through the mouse brainstem, midbrain, and diencephalon (Rhomb, Mes, Pretectum, Thalamus, Prethalamus), as well as a separate section through the telencephalic vesicle divided into Subpallium (lateral ganglionic eminence, LGE) and Pallium (thick black dash lines delimit the LGE from the cortical field -Cx, Hipp, DG- and from the pallial amygdala field -Amygd). The thick red line in the brainstem and rostral forebrain is the longitudinal alar-basal boundary (note the entire telencephalon is alar). The proliferative ventricular zone (with dotted mitotic cells) is uniformly colored in blue; the ventricular cavities of the brain appear in solid black. Sometimes there is an additional proliferative deep stratum called “subventricular zone,” here colored were present in green. The alar mantle zone with differentiating neurons is labeled generally in yellow, though at some superficial places local cortical plates are distinguished in red, orange, or violet. The basal mantle appears partially colored in pink. Pairs of thin black dash lines identify particular sectors of the brain wall (Cb, Mes, SC, Thalamus, Subpallium, Cx), and the associated black arrows attract your attention to auxiliary schemata showing the variant radial structure obtained at the respective sites, sometimes showing a full cross-section across all dorsoventral zones, otherwise only selected alar plate sites. It depends on the region represented, as telencephalon (pallium, subpallium), prosomere 2 (thalamus, habenula, basal plate) mesencephalon, and cerebellum. Taken from Rubenstein and Puelles (2004; their Figure 4).

**Figure 5:**
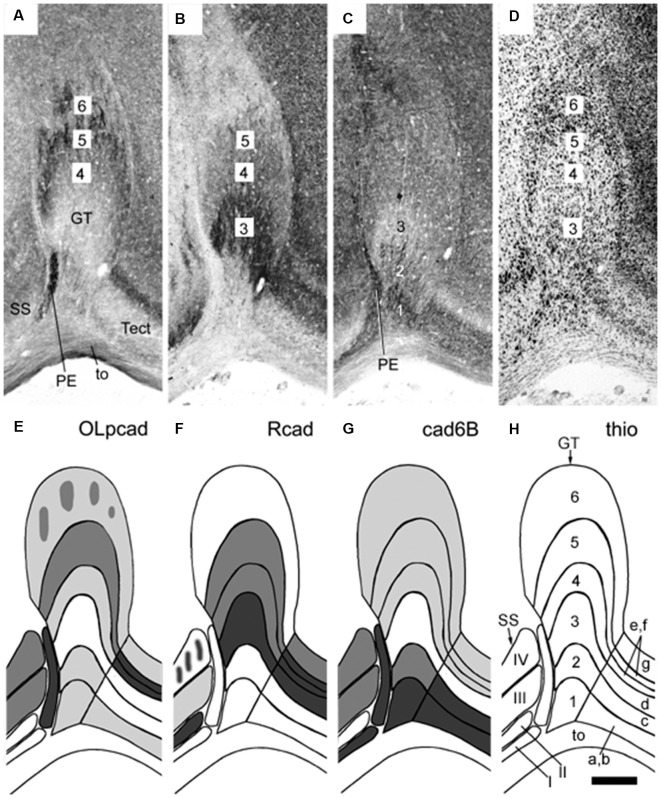
Panels **(A–H)** illustrating stratified differential expression of three cadherins (OLpcad, Rcad, and cad6B in **A–C**; diagrams in **E–G**) compared to Nissl stain (thio; **D**, the diagram in **H**) in the avian (chicken) griseum tectale (GT). This is an alar mesencephalic retinorecipient FMU found rostral to the midbrain optic tectum (Tect; tectal layers a–g marked in **H**) and caudal to superficial partly layered pretectal nuclei (PE, SS; layers I-IV; see map in **H**). The Nissl stain reveals six distinct GT layers deep to the optic tract (to; layers 1–6), with presumably different neuronal types populating each of them. Variant cadherin expression (**A–C**; schemata in the lower panels) seems associated with neurons forming particular deep or superficial strata. From Müller et al. (2004; their Figure 6).

Neurogenesis occurring in these microzonal unit domains causes production and accumulation of immature neuronal derivatives of specific sorts (types) in the local mantle layer. The latter expands between the ventricular zone and the pial surface, in a growing texture of pronuclei (immature cell masses) held together by ventricular cell processes and radial glia cells (Figures 2, 3). The interplay of a variety of adhesive properties (coded by the different genetic profiles) leads gradually to definitive nuclei or other neuronal arrangements (reticular, cortical; Figures 4, 5). We frequently observe that final nuclear derivatives appear distributed into periventricular, intermediate, and superficial strata of the mantle at postnatal stages, though each histogenetic domain may show peculiarities in this aspect (Figure 4; e.g., lack of a periventricular stratum in the adult cerebellum).

### Radial Unit/FMU Development

The first steps of neighbor-independent radial unit histogenetic activity are the processes of *proliferation (surface and thickness growth), neurogenesis*, and *vascularization* (see in this last regard Puelles et al., 2019). Heterochronic patterns of proliferation and neurogenesis are specified locally within the unitary neuroepithelial areas in ways still hardly investigated (Puelles et al., 1987). Regional growth differences within and between neighboring units give rise to morphological phenomena of the neural wall such as vesiculation (local expansion) and constrictions (local stagnation of growth, Figure 2B; Crossley et al., 2001; Echevarría et al., 2003; Vieira et al., 2010). Once the progenitor cells start producing daughter cells that stop proliferating and detach from the ventricular zone to differentiate as neurons, there begins the physical formation of the radial dimension in the central nervous system (Figures 2A,B). Note the produced neuronal type may change over time in the same FMU, so to speak “in successive neurogenetic waves” (Figure 2B), or may vary in a “salt and pepper” pattern involving mutual lateral inhibition phenomena. The changing radial dimension is correlated temporo-spatially in a stereotyped pattern along with the whole set of FMUs (Puelles, 2001, 2013; Garcia-Calero, 2005; Puelles et al., 2008; Garcia-Calero et al., 2020). This means that a uniform type, a mixture of types, or a sequence of types, of immature neurons, originated from a given molecularly defined FMU migrate within the mantle zone inside-out (eventually, translocating only their somata), or accumulate passively outside-in along the radial dimension. The local radial glial scaffolding guides cellular distribution within the respective mantle layer, where the differentiating neurons and glia cells variously aggregate, stratify or disperse (Figure 3; Rakic, 1972, 1974; Puelles, 2001, 2018; Garcia-Calero, 2005; Puelles et al., 2008).

The molecularly defined ventricular zone, patch, or area, the corresponding mantle layer (containing the neuronal/glial derivatives and incipient vascular elements), and the corresponding pial surface, all interconnected by radial glia processes, build the *radial histogenetic units* or FMUs (Figures 2, 3). The *role* as FMUs of such units arises because each of them contributes as a plastic morphogenetic element an essential block of the whole anatomic edifice of the adult brain (Figure 1C; Nieuwenhuys and Puelles, 2016). They are “radial” because each FMU extends as a polyclone from the ventricular surface to the pial surface (Figure 2). They are specific *unrepeated* units because of their unique molecular profiles, which also represent in potency their specific prospective fates in their respective epigenetic contexts (Figure 4). The “new neuromorphology” postulates that causal explanation of the final brain structure and shape (or of any of its subregions, such as e.g., the hypothalamus, or the amygdala) is not possible without a theory jointly contemplating the unique or shared developmental phenomena occurring within the whole set of relevant radial FMUs. Brain parts having only one FMU, or just a few similarly structured FMUs, are “simple” to explain and understand from a typical partial example (case of the neural retina and cerebellum), whereas regions having many FMUs are as “complex” as the number of FMUs and their respective differential behavior may impose. Nevertheless, such complexes remain amenable to analysis as long as we identify first the involved FMUs and their differential properties (see the case of the pallial amygdala below).

The immature neurons may move radially or tangentially within the histogenetic radial units (or stay put where they are), following different patterns. Such displacements may be reflected ultimately as inside-out or outside-in arrangements of older vs. younger neurons (e.g., the inside-out mammalian isocortex; Angevine and Sidman, 1961; Rakic, 1974; Bayer and Altman, 1991; Noctor et al., 2004 vs. the outside-in mammalian thalamus; McAllister and Das, 1977; Altman and Bayer, 1989). Histogenetic options, in the form of nuclear, layered, or reticular disposition of single or mixed cell types in the mantle stratum of the radial units, are related to multiple combinations of more or less shared (or complementary) adhesion molecules and receptor expression at the surface of the cells. This includes, for example, the large family of cadherin adhesion molecules, which depend on calcium and form homotypic attachments (Figure 5; Yoon et al., 2000; Redies et al., 2001; Stoya et al., 2014: Müller et al., 2004).

**Figure 6:**
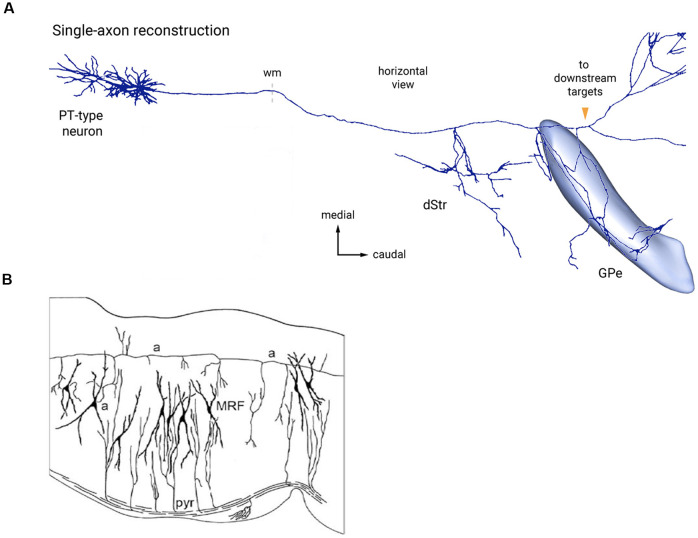
Two examples of bypassing axons producing selective collateral branches at specific FMUs. **(A)** Reconstruction of single cortical pyramidal cell axon (PT-type neuron) projecting to the thalamus and other targets in the brainstem. It produces selectively collaterals for the dorsal striatum (dStr) and the external globus pallidus (GPe), which each represent parts of different subpallial FMUs. Copied with permission from Abecassis et al. (2020; part of their Figure 6A). **(B)** Sagittal Golgi-impregnated section through the hindbrain reticular formation of the rat (MRF), showing collaterals of the ascending axon (a-a) of cell “a” at different rostrocaudal rhombomeric FMU sites. Similar for FMU-restricted successive collaterals of the descending pyramidal tract (pyr). Taken from Nieuwenhuys and Puelles (2016), plate 123.

Finally, neuronal survival depends on securing appropriate trophic support through connectivity, using axonal navigation to find suitable targets, resulting in efficient synapsis formation with functionally adapted afferents and targets. Such connectivity patterns are controlled by differential genomic readout by each cell type, as a part of their differentiation within the FMU, and make use of the local combinatorial molecular profile. As a result of such differentiation, the axonal growth cone membrane, as well as the dendritic or somatic cell membranes, acquire differential molecular constituents (epitopes). This confers to the types of synaptic contact they establish specific interactive functional properties acted upon over time by natural selection (producing adaptive fitness). Connections can be established both between different neurons of the same *radial histogenetic unit* and with neurons of neighboring or distant units. Interestingly, axons seem to be able to distinguish when they pass from one radial unit into another. Some visibly produce collaterals at each unit crossed, or at specific non-sequential units, or generate them *only* when they reach the main targeted unit (Figure 6). How they do this selecting trick remains obscure (possibly there are local differential signals in the intercellular matrix of each FMU to which axons are sensitive, or there is a local response of the axonal growth cone membrane towards discontinuities in the molecular cell surface epitopes encountered on the *glial processes* of the different areas). Selective navigation of axons through permissive local environments, and the formation of trophic functional synapses, stabilize the hodological neural structure that interconnects FMUs (Puelles et al., 2008). In this sense, connectivity patterns also depend on relative neurogenetic timing, since depending on its birthdate each neuronal type has a particular sequence of migration and differentiation which opens specific windows of opportunity for establishing correct (biologically useful) connections (Bayer and Altman, 1987).

In conclusion, the neural *radial histogenetic units* or FMUs represent relatively independent histogenetic micro complexes of the brain wall. They necessarily interact among themselves in a complex way, not only hodologically, but also mechanistically, by the forces created within and between the units by their growth, which must equilibrate into final adult shapes. These FMU units show a clear-cut spatial organization in which all generic histogenetic mechanisms described in neural development occur in subtly unique ways, even where neighboring units share various properties and behave similarly. Because of shared properties, most radial units tend to organize into periventricular, intermediate, and superficial strata, which may variously mature as nuclear, cortical, or reticular cell formations. Apart from developing their own inner circuitry, which produces, so to speak, their own (potentially unique) functional algorithm, individual FMUs gradually interconnect with a specific network of other FMUs, thus forming part of larger neural functional systems.

Tangential migration occurs where neuronal populations move orthogonally to local radial glia. This may occur less importantly inside a radial unit, but acquires added relevance when cells born in one radial unit translocate tangentially into other FMUs; this may occur along the dorsoventral or anteroposterior directions (sometimes a sequence of both) and may be selective or indiscriminate as regards the targeted areas. For instance, the basilar pontine nuclei form after a *selective* two-step tangential migration from the rhombic lip, while diverse sets of GABAergic subpallial interneurons migrate tangentially *indiscriminately* into most cortical and nuclear pallial areas. The migrated cells incorporate evolutionarily into the local circuitry of the receiving FMU, presumably under natural selection of resulting improvements in biological fitness. Such tangential migrations are much more common and varied in type than was initially thought 50 years ago (e.g., see some hypothalamic ones reported by Díaz et al., 2015). This property to acquire ectopic fates (perhaps analogous in some molecular aspects to the rich connective properties of axons) adds complexity to the neural histogenetic program (Marín and Rubenstein, 2001; Puelles, 2001; Garcia-Calero, 2005; Puelles et al., 2008; Murillo et al., 2015; Nieuwenhuys and Puelles, 2016; Ruiz-Reig et al., 2018).

## Amygdalar Models in Mammals

### Amygdalar Non-radial Models

The amygdala is a complex nuclear structure located at the tip of the temporal pole in mammals. It is implicated in emotion learning and processing (Burdach, 1819–1822; Johnston, 1923; Weiskrantz, 1956; De Olmos et al., 1985, 2004; Alheid et al., 1995; Swanson and Petrovich, 1998; Amaral et al., 2003; Phelps and LeDoux, 2005; LeDoux, 2007; Martínez-García et al., 2012; Rolls, 2014, 2015; Olucha-Bordonau et al., 2015). Burdach (1819–1822) first described it as an almond-shaped structure present in the mantle layer of the temporal human pole; he referred actually to the modern basolateral complex (BL). Johnston (1923) extended this definition to nuclei surrounding the BL, including corticoid masses at the telencephalic surface. Ulterior cytological/immunohistochemical, connectivity and developmental studies subdivided the amygdala mainly into a pallial basolateral/basomedial (BM) complex and a subpallial centromedial complex. Various corticoid masses appear at the amygdalar brain surface, some of which receive olfactory and vomeronasal projections. Transitional amygdalo-cortical areas, such as the pallial amygdalo-piriform (APir) and amygdalo-hippocampal (AHi), or the subpallial intercalated cell masses, were also added ulteriorly to these models (Swanson and Petrovich, 1998; Martínez-García et al., 2012; Olucha-Bordonau et al., 2015; Medina et al., 2017).

#### Cyto/Chemoarchitectonic Amygdalar Models

Studies of amygdalar structure generally identified the individual nuclei or corticoid formations based on their topography in conventional coronal sections, often grouping them in relation with cytochemical data (see Table 1 on Topographical models: pallial derivatives of the amygdalar region; Johnston, 1923; Krettek and Price, 1978; Turner and Zimmer, 1984; De Olmos et al., 1985, 2004; Alheid et al., 1995; Martínez-García et al., 2012). A consensus model gradually emerged, which described in mammals a phylogenetically primitive central nuclear complex surrounded by supposedly new amygdalar nuclei; a medial nuclear group located in the medial wall of the temporal pole; a basolateral nuclear group (lateral, basal, and accessory basal, later called basomedial) deep to the corticoid structures; and various superficial corticoid masses (anterior, posterolateral, posteromedial). These nuclei were named with regard to their apparent topographical disposition in coronal sections, attending to Nissl staining and chemoarchitectural/immunohistochemical profile (see, for example, the cytoarchitectonic description of the rat and cat amygdala in Krettek and Price, 1978; the cytoarchitectonic and chemoarchitectonic study based, for example, upon acetylcholinesterase activity or the Timm method in the rat amygdala by De Olmos et al., 2004). There was little or no reference to the pattern of local histogenesis, which was widely assumed to represent mostly radially migrated populations, without specifying the ventricular loci; most non-periventricular populations (i.e., intermediate and superficial strata elements) were not ascribed to specific progenitor domains or histogenetic strata.

**Table 1:** The radial unit, subunits and nuclei in the *pallial amygdalar radial model* (Garcia-Calero et al., 2020), and pallial amygdalar complexes (lateral, basolateral, and basomedial,) and nuclei, together with corticoid masses in the *topographical amygdalar models* (Krettek and Price, 1978; De Olmos et al., 1985, 2004; Alheid et al., 1995; Paxinos and Franklin, 2007, 2013).

Radial amygdalar model			Topographical amygdalar models	
Radial units	Radial subunits	Nuclei	Nuclei	Complexes
*Lateral*		L, LI, CxAR	LaVM, LaVL, LaDL	Lateral
*Basal*	*Basolateral*	BLP, BLA, BLI, CxAC	BLP, BLA, BLV	Basolateral
	*Basomedio-medial*	BMPM, BMIM, PLCoR		
	*Basomedio-lateral*	BMPL, BMIL, PLCoC		
*Anterior*		BMA, ACo	BMA, BMP	Basomedial
*Posterior*	*Rostro-lateral*	AHiRL, PMCoRLi, PMCoRLs	ACo, PLCo, PMCo	Corticoid masses
	*Rostro-medial*	AHiRM, PMCoRMi, PMCoRMs		
	*Caudo-lateral*	AHiCL, PMCoCLi, PMCoCLs		
*Retro-endopiriform*		REP, REPI, REPCo		

*CxA and AHi nuclei are not included in the topographical amygdalar model due to their disposition as cortical-amygdalar transitional areas. Abbreviations: ACo, anterior cortical nucleus; AHiCL, amygdalo-hippocampal area, caudolateral part; AHiRL, amygdalo-hippocampal area, rostrolateral part; AHiRM, amygdalo-hippocampal area, rostromedial part; BLA, anterior basolateral nucleus; BLI, intermediate basolateral nucleus; BLP, posterior basolateral nucleus; BLV, ventral basolateral nucleus; BMA, anterior basomedial nucleus; BMP, posterior basomedial nucleus; BMIL, lateral intermediate basomedial nucleus; BMIM, medial intermediate basomedial nucleus; BMPL, lateral posterior basomedial nucleus; BMPM, medial posterior basomedial nucleus; CxA, cortex-amygdala transitional area; CxAC, cortex-amygdala transitional area, caudal part; CxAR, cortex-amygdala transitional area, rostral part; L, lateral nucleus; LaDL, lateral amygdalar nucleus, dorsolateral part; LaVL, lateral amygdalar nucleus, ventrolateral part; LaVM, lateral amygdalar nucleus, ventromedial part; LI, intermediate lateral nucleus; PLCo, posterolateral cortical nucleus; PLCoC, posterolateral cortical nucleus, caudal part; PLCoR, posterolateral cortical nucleus, rostral part; PMCo, posteromedial cortical nucleus; PMCoCLi, posteromedial cortical nucleus, caudolateral part, intermediate stratum; PMCoCLs, posteromedial cortical nucleus, caudolateral part, superficial strataum; PMCoRLi, posteromedial cortical nucleus, rostrolateral part, intermediate stratum; PMCoRLs, posteromedial cortical nucleus, rostrolateral part, superficial stratum; PMCoRMi, posteromedial cortical nucleus, rostromedial part, intermediate stratum; PMCoRMs, posteromedial cortical nucleus, rostromedial part, superficial stratum; REP, retroendopiriform nucleus; REPI, retroendopiriform intermediate area; REPCo: retroendopiriform cortical area*.

The basolateral nuclear group was subdivided first in lateral, basolateral, and basomedial complexes, attending in the naming again to their mutual topographical relationships, in which the descriptor *basal* indicates “*topographically ventral”*, or “*closer to the ventral surface”*. These first basolateral divisions were split later into anterior (topographically rostral levels), posterior (topographically caudal levels), and dorsal or ventral nuclei depending on the disposition in coronal sections (most of these studies were developed in coronally sectioned rodent brains, which are oblique to the orientation of amygdalar radial glia; Remedios et al., 2007; Garcia-Calero et al., 2020). Examples of this nomenclature are the basolateral anterior and basolateral posterior nuclei (BLA, BLP; Table 1; Figures 36–57 in Paxinos and Franklin, 2007), the basomedial anterior and basomedial posterior nuclei (BMA, BMP; Table 1; Figures 36–52 in Paxinos and Franklin, 2007), the dorsolateral, ventrolateral and ventromedial subdivisions in the lateral complex (LaDL, LaVL, LaVM; Table 1; Figures 41–49 in Paxinos and Franklin, 2007), as well as the dorsal and ventral division of the medial amygdala, with anterior and posterior subdivisions (MeAD, MeAV, MePD, MePV; Figures 37–49 in Paxinos and Franklin, 2007). The olfacto-recipient superficial corticoid masses were named again in relation to anterior, postero-lateral, posteromedial coronal topographic coordinates (ACo, PLCo, PMCo; Table 1; Figures 33–62 in Paxinos and Franklin, 2007). This standard, or consensus, rodent nuclear amygdalar terminology is found widely in textbooks, atlases, and experimental studies, but divides arbitrarily some radially arranged amygdalar structures into *anterior* superficial parts, some vaguely characterized intermediate nuclei, and *posterior* periventricular nuclei. This was historically an *unnoticed* consequence of disregarding the easily observable fact (in sagittal sections) that most amygdalar nuclei lie *rostral* to their correlative ventricular surface. There is an obliquity of 45 degrees between the natural (histogenetic) amygdalar radial dimension and the standard coronal sections (widely assumed to be orthogonal to an ideal, non-molecularly defined forebrain axis ending in the telencephalon; Herrick, 1910; Swanson, 2012).

#### Functional Amygdalar Models

Another option followed in the literature was to classify amygdalar nuclei into functional regions according to neurotransmitter content and connectivity patterns (Johnston, 1923; Krettek and Price, 1978; De Olmos et al., 1985, 2004; Alheid et al., 1995; Swanson and Petrovich, 1998; McDonald, 2003; Sah et al., 2003). In this sense, the main amygdalar subdivisions distinguished were the *basal-ganglia-like* (or *striatum-like*) structures, meaning the classic medial and central amygdala with the intercalated nuclei and the extended amygdala added, and the *cortex-like* structures, which would encompass the BL and associated corticoid masses. For example, Swanson and Petrovich (1998) emphasized in an influential review the separation of striatum-like and cortex-like moieties of the amygdala based on the predominance of GABAergic vs. glutamatergic cell populations, respectively (notably, this agrees with the developmental distinction of subpallial and pallial moieties). Functional grouping of amygdalar structures also caused the separate treatment of the superficial corticoid structures, as parts of the main or accessory olfactory systems. The lateral and basal deep nuclei entered in separate amygdalar blocks related to thalamic and associative cortical inputs (Krettek and Price, 1978; De Olmos et al., 1985, 2004; Swanson and Petrovich, 1998; Sah et al., 2003; Martínez-García et al., 2012; Olucha-Bordonau et al., 2015; Medina et al., 2017). Remarkably, some superficial and deep amygdala neurons share similar cell types (e.g., Golgi studies of Hall, 1972).

#### Developmental Amygdalar Models

Johnston (1923), taking a comparative viewpoint, proposed a bipartite subdivision in the amygdala, held to contain a putative *ancient part* enclosing the central and medial nuclei, and a secondarily evolved *new region* that would encompass the lateral, basal, and corticoid areas; there was, also, a subdivision into subpallial and pallial amygdala (Holmgren, 1925). Embryological studies first distinguished pallial and subpallial subdivisions in the amygdala, which implicitly are large primary radial regions (Holmgren, 1925; Kuhlenbeck, 1973; Smith-Fernandez et al., 1998; Puelles et al., 2000, 2016a; Medina et al., 2004, 2017; Tole et al., 2005; García-López et al., 2008). The central and medial amygdalar nuclei were often considered a part of the molecularly defined subpallial amygdala (Puelles et al., 2000; García-López et al., 2008; Martínez-García et al., 2012; Medina et al., 2017), as had been proposed likewise by chemoarchitectonic studies (Swanson and Petrovich, 1998). The *tetrapartite pallial model* (Puelles et al., 2000) proposed the subdivision of the pallial amygdala in *lateral* and *ventral* pallial subregions. The ventropallial domain was subsequently held to include the lateral (La) and BM complexes, jointly with the ACo and AHi /PMCo areas, whereas the lateral pallium was deduced to represent the embryonic source of the BL and the PLCo (Puelles et al., 2000; Medina et al., 2004). Later analysis of the selective lateropallial claustral and endopiriform expression marker *Nr4a2* (an orphan receptor), indicated that the pallial amygdala does not contain a lateral pallium subdivision (Puelles, 2014; Puelles et al., 2016a,b).

The *Dbx1* gene seems to be expressed selectively in the ventricular zone of the mammalian ventral pallium subdivision (Yun et al., 2001; Medina et al., 2004; Puelles et al., 2016b). Initial progeny analysis of neuronal derivatives from the *Dbx1*-positive ventricular patch identified labeled projection neurons in the basolateral and corticoid amygdalar masses, consistent with the notion that these neurons originate from a ventropallial source (Hirata et al., 2009; Waclaw et al., 2010). A more detailed progeny analysis performed in coronal sections by Puelles et al. (2016b) concluded that ventral pallium derivatives might include the anterior parts of the basolateral and basomedial complexes, but not their posterior (i.e., periventricular) parts, unless a *Dbx1*-negative ventricular patch of ventral pallium was postulated (to explain positive and negative labeled parts of given nuclei). Alternatively, the caudal parts of this complex might be ascribed to a new pallial subdivision, the *ventrolateral caudal pallium*, which would be restricted in its extent to the caudal periventricular amygdala (Puelles et al., 2016b; see also Desfilis et al., 2018); this solution is inconsistent with the notion of obliquely oriented radial histogenetic units given above. Also, AHi was described as a derivative of the medial pallium in Abellán et al. (2014). In contrast to these concepts, Puelles et al. (2019) proposed recently an updated *cortical ring model*, which questioned a necessary one-to-one causal relationship of *amygdalar* pallial parts with *cortical* pallial subdivisions. They suggested instead that the pallial amygdala should be considered an independent non-cortical complex structure (consisting of several FMUs) located *external* to the cortical pallium field, irrespective of sharing some gene markers with it. The cortical field results from the sum of the cortical ventral, lateral, dorsal, and medial pallium sectors arranged into a central dorsopallial island surrounded by mesocortical and allocortical peripheral rings.

In addition to pallial/subpallial radial subdivisions in the amygdalar complex, developmental tangential migration into the amygdala has been variously reported. Wang and Lufkin (2000) and García-Moreno et al. (2010) described Otp cells migrating tangentially from the hypothalamic paraventricular area into the medial amygdala. These seem to be accompanied by *Sim1*-expressing cells, which invade selectively the pallial amygdala (Garcia-Calero et al., submitted). A variety of subpallium-derived cell populations migrate tangentially into the pallial amygdala (Waclaw et al., 2010; Medina and Abellan, 2012; Puelles et al., 2016c). Also, Remedios et al. (2007) observed a caudally originated tangential migration of pallial cells (termed ‘caudal amygdaloid stream), which forms the nucleus of the lateral olfactory tract (NLOT) within the subpallial anterior amygdala. These results point to a heterogeneous cell type composition of various amygdalar regions, with at least pallial and subpallial subdivisions, and hypothalamic and various subpallial tangentially migrated cell types.

### Amygdalar Radial Models

**Figure 7:**
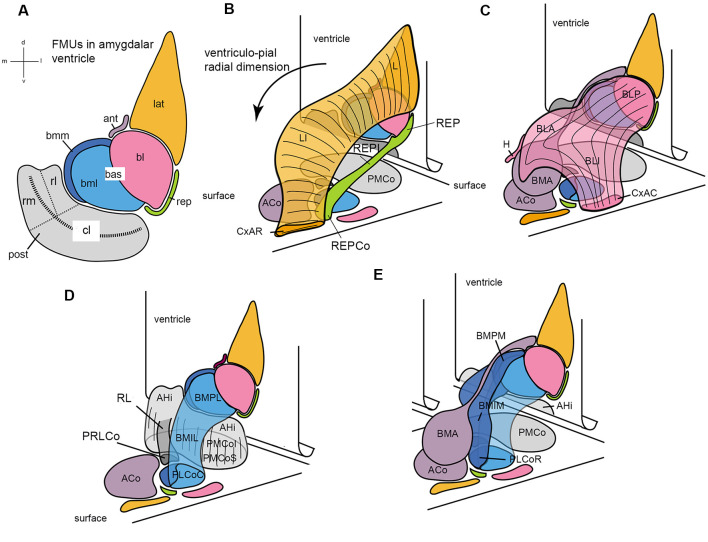
Set of diagrams attempting to represent the packet of adjacent radial units recently identified in the mouse amygdala (Garcia-Calero et al., 2020; their Figure 2). Panel **(A)** is a flattened map of the amygdalar ventricular surface, which lies largely caudal to the amygdala and bends backwards into the hippocampal cortex at the bottom, roughly at the middle of the posterior unit, along the curved striped line. The diverse pallial amygdalar FMUs are identified in the adult brain at their ependymal end by a color code that will be used likewise in the remaining panels, as well as by partly conventional topographic abbreviated names (lateral, lat; anterior, ant; basolateral, bl; basomediolateral, bml; basomediomedial, bmm; retroendopiriform, rep; posterior, post; the large “post” unit subdivides into small rostrolateral “rl” and rostromedial “rm” parts, and a larger caudolateral “cl” part). The ventricular bending in the middle of the post reappears represented tri-dimensionally in panels **(B–E)**, in which the amygdalar ventricular surface with the colored FMUs traced on it is represented schematically as a vertical plane in perspective that ends bending backward at its ventral end. The corresponding amygdalar pial surface (marked “surface”) is represented realistically as a plane in perspective located under the amygdalar complex, at a right angle with the ventricular surface. It can be seen that the map of radial units represented at the pial surface corresponds one-to-one to the colored ventricular areas, though the shapes at the surface are different. A black arrow in **(B)** identifies the local ventriculo-pial radial dimension. Panels **(B–E)** illustrate each two or three of the cited, color-coded radial units in a partial 3D representation, designed to clarify collectively the visualization of how each unit relates to the others (and to the ventricular and pial surfaces). Panel **(B)** shows the orange lat unit jointly with the underlying green rep unit, at the lateral end of the amygdalar complex. The lat consists of the deep lateral nucleus (L), the intermediate lateral nucleus (LI), and the superficial rostral cortico-amygdalar transition area (CxAR). The rep consists of the deep retroendopiriform nucleus (REP), the intermediate REP (REPI), and the superficial corticoid REP (REPCo). **(C)** Reconstructs the pink bl unit together with the underlying mauve ant unit. The bl contains the deep basolateral posterior nucleus (BLP), the intermediate basolateral anterior and basolateral intermediate nuclei (BLA; BLI), and the superficial caudal cortico-amygdalar transition area (CxAC). The ant is singular in having no deep adult element (only it’s set of radial glia reach the ventricle) since its progeny accumulates within the intermediate basomedial anterior nucleus (BMA) and the superficial anterior cortical nucleus (ACo), found medially to the orange CxAR (see ACo full surface area in D). The overall shape of the ant with its ependymal glial tail is seen again in **(E)**. **(D)** Shows a 3-D reconstruction of the gray post unit, compared to the light blue bml unit. The post in general consists of the periventricular amygdalo-hippocampal area (AHi) and the superficial posteromedial cortical nucleus (PMCo), which contains the equivalent of the intermediate and superficial strata (the latter labeled PMCoS); note also the distinct “rl” part of the post (RL; in darker gray color), which ends superficially separately in its own cortical patch (PRLCo behind the ACo); the light gray “rm” part of post can be seen behind RL, whereas the bigger caudolateral end of the post appearing from behind the light blue bml unit is the “cl” part. The bml unit is a bent cylinder that contains a deep lateral basomedio-posterior nucleus (BMPL), the intermediate lateral BM nucleus (BMIL), and has its superficial end at the caudal part of the classic posterolateral cortical nucleus (PLCoC). **(E)** Illustrates jointly the light blue bml unit and the medially associated dark blue-colored bmm unit, together with the mauve ant unit. Both basomedial elements are intimately related at the ventricular surface (bmm and bml in **A**) and the pial surface (PLCo); they form a basomedial radial complex, which jointly with the pink bl **(C)** forms a large basal complex. The bmm consists of the deep medial basomedio-posterior nucleus (BMPM), the intermediate BMIM, and the rostral part of the PLCo (PLCoR). Conventional versions of amygdalar pallial nuclei mix the ant BMA with the BMP of the basomedial complex (error now clarified by distinct molecular patterns) and do not postulate any superficial ends for both the bl and lat units.

A first attempt to organize the amygdala along the radial dimension was published by McDonald (2003) based in Stephan and Andy’s (1977) amygdalar studies in insectivores and primates. These authors described the cortico-basolateral group as a radial entity characterized by similarly sized neurons and indistinct boundaries between cortical and inner structures. PLCo at the brain surface relates to BMP in the periventricular zone, subpial PMCo connects radially with the periventricular AHi and the ACo corticoid mass groups with BMA (McDonald, 2003). We largely corroborated these results in our more detailed *pallial amygdala radial model*, which presented additionally several novel concepts, involving previously unrecognized intermediate and superficial nuclei, as well as a rich assortment of new molecular data (Table 1; Figure 7; Garcia-Calero et al., 2020). McDonald (2003) observed different neurogenetic patterns between some pallial amygdalar areas (with an outside-in pattern) and surrounding cortical structures such as piriform or hippocampal areas (with an inside-out neurogenetic pattern). Garcia-Calero et al. (2020) reviewed neurogenetic data in the literature and found they unanimously support the suggested histogenetic position of the pallial amygdala outside (independent from) the piriform-entorhinal-hippocampal allocortical ring, as was contemplated in the *cortical ring model* (Puelles et al., 2019).

#### Pallial Amygdalar Radial Model

In this model, the authors investigated at late embryonic and postnatal to adult stages the theoretical possibility of rearranging the conventional coronal-topographic array of amygdalar nuclear groups into a set of credible radial units (Figure 7; Garcia-Calero et al., 2020). We assumed that true radial units should have specific packets of radial glia unifying their superficial, intermediate, and periventricular elements (Figure 3). Experimental DiI labeling of the subpial endfeet of radial glia checked whether given superficial and intermediate elements indeed appear linked to specific periventricular structures. We also tested in this way whether any part of the piriform cortex lies topologically *superficial* to any amygdalar component. As expected from earlier topologic analysis (Puelles et al., 2019), the conclusion was that the pallial amygdala lies *adjacent*, but not deep, to the piriform cortex (including the amygdalo-piriform area, which behaves as a part of piriform cortex), and it maintains similar neighborhood relationships concerning the hippocampal and entorhinal cortex (Puelles et al., 2019; their Figure 10). In principle, accordingly, the expected amygdalar radial FMUs should contain in a stratified pattern the cortical, lateral, and basal components, as well as the amygdalo-hippocampal complex, which behaves as an amygdalar pallial part. Due to the multiplicity of described distinct amygdalar nuclei, plus the various chemoarchitectonic and functional properties, we expected that multiple FMUs probably jointly construct developmentally the pallial amygdala. These units would be expected to show differential molecular profiles.

Radial glia studies using descriptive immunochemical analysis during mouse development (e.g., Remedios et al., 2007) already indicated that the amygdalar radial dimension does not agree with the standard coronal section plane. It corresponds in sagittal sections rather to an *oblique plane* relative to the cortical back of the brain, or the plane of the caudal ventricular cavity, which is roughly parallel to the surface of the entorhinal cortex. The standard coronal sections found in rodent atlases systematically cut all amygdalar radial glia trajectories into pieces. An oblique radial amygdalar section plane seems thus necessary for visualizing whole (or nearly so) the radial domains in the amygdalar region. We used this *ad hoc* methodological approach to study pallial amygdalar radial complexes characterized by differential chemo- and genoarchitectonic patterns.

There are five main radial units, or macrounits, called *lateral*, *basal*, *anterior*, *posterior*, and *retroendopiriform* units, all stratified in principle into periventricular, intermediate, and superficial strata (Table 1; Figure 7; Garcia-Calero et al., 2020). Also, the *basal* and *posterior* radial units are each subdivided into three subunits, according to changing areal molecular properties. This creates a total of nine* molecularly distinct pallial amygdalar FMUs*, each in principle with three strata, raising to a novel high the notion of amygdalar complexity (nine times three strata give 27 systematically distinguishable structural items; some 80 gene markers supported this schema). However, the simpler division into five main or macro-radial units (Figure 7) is helpful for comparisons with the conventional amygdalar schema (Table 1).

**Figure 8:**
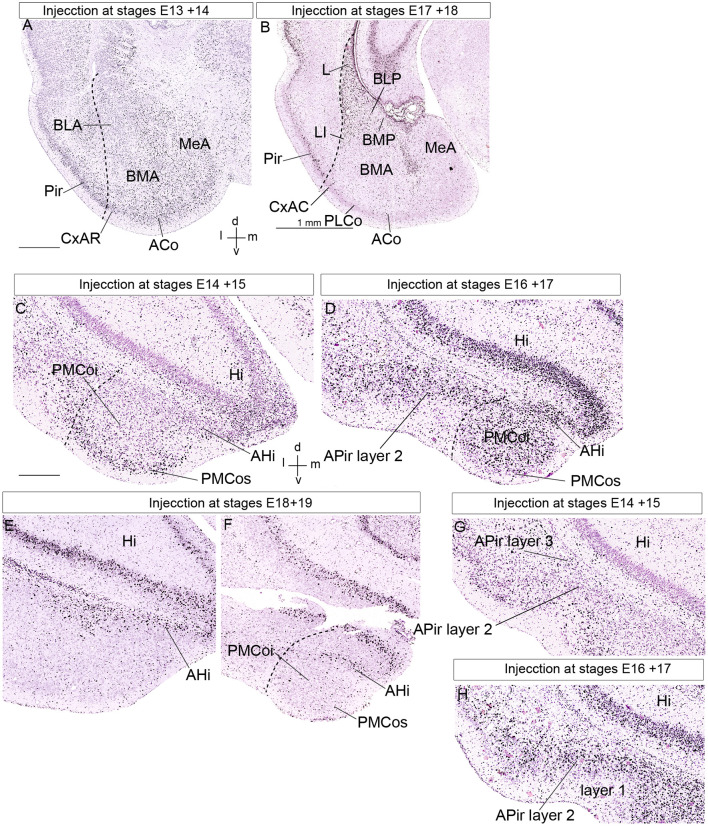
Public 3H-thymidine autoradiography images from birthdate analysis in rat embryo brains obtained from https://braindevelopmentmaps.org/ (Bayer S, and Altman J). The pregnant rats were injected at two successive days at the embryonic times indicated, and the offspring were sacrificed postnatally at P5 and processed autoradiographically on the slide. Spatial orientation is indicated at the bottom right-hand corner in panels **(A,C)**. **(A,B)** Illustrate the neurogenesis pattern (unlabeled already born cells vs. strongly labeled recently born cells) at the lateral, basal, and anterior amygdalar radial units. Superficial cells are born earlier than periventricular cells. **(C–F)** Show the neurogenesis pattern at the posterior amygdalar radial unit. Likewise, early-born cells are located at the superficial PMCo, whereas later-born cells appear at the periventricular AHi; the complex is produced as a whole between E16 and E19. In contrast, panels **(G,H)** show an inverted inside-out pattern of neurogenesis at the non-amygdalar APir olfactory cortical subarea. **(C,G)** Are images of the same section, in panel **(C)** we focused our attention upon the AHi deep post region, whereas attention was focused on the APir region in panel **(G)**. Abbreviations: ACo, anterior cortical nucleus; AHi, amygdalo-hippocampal area; APir layer 1, amygdalo-piriform area, layer 1; APir layer 2, amygdalo-piriform area, layer 2; APir layer 3, amygdalo-piriform area, layer 3; BLA, anterior basolateral nucleus; BLP, posterior basolateral nucleus; BMA, anterior basomedial nucleus; BMP, posterior basomedial nucleus; CxAR, cortex-amygdala transitional area, rostral part; Hi, hippocampus; L, lateral nucleus; MeA, medial amygdala; Pir, piriform cortex, PLCo, posterolateral cortical nucleus; PMCoi, posteromedial cortical nucleus, intermediate stratum; PMCos, posteromedial cortical nucleus, superficial stratum. The scale bar represents 500 μm.

The *lateral* radial macrounit contains the classical lateral nucleus (LI) subdivided into periventricular and intermediate parts (L, LI respectively); superficially, it newly includes the rostral cortex-amygdala transitional area (CxAR; L, LI, CxAR; Figures 7A,B, 8A,B; Table 1). The CxAR was previously ascribed to the piriform cortex.

The *basal* radial macrounit contains the conventional *basolateral* and *basomedial* portions, which encompass the classical BLP and BMP nuclei within the periventricular stratum. These end superficially at the caudal cortex-amygdala transition area (CxAC) and the PLCo, respectively (BLP, BMP, CxAC, PLCo; Figures 7A,C–E, 8A,B; Table 1). However, the *basomedial* portion unexpectedly turned out to be subdivided molecularly into parallel lateral and medial parts (the *basomediomedial* and *basomediolateral* subunits; *bmm*, *bml*) whose periventricular components are named similarly (BMPM; BMPL); these radial subunits end superficially at the caudal and rostral halves of PLCo, respectively (PLCoC, PLCoR; 7A,D,E; Table 1). We discovered various novelties in the *basal* intermediate stratum. The intermediate stratum of the *basolateral* subunit mainly consists of the classic BLA, together with a novel more superficial cell mass leading into the superficial CxAC, the intermediate basolateral nucleus (BLI). On the other hand, the intermediate stratum of the *bmm* and *bml* subunits is formed by the likewise novel lateral intermediate basomedial (BMIL) and medial intermediate basomedial (BMIM) nuclei (BLA, BLI, BMIL, BMIM; Figures 7A,C–E, 8A,B; Table 1). Differential molecular profiles strongly support these new subdivisions (Table 1).

The *anterior* radial macrounit exceptionally concentrates all its progeny in the intermediate and superficial strata (Garcia-Calero et al., 2020; Garcia-Calero and Puelles, submitted); it contains the classical BMA nucleus in the adult intermediate stratum and superficially the ACo (BMA, ACo; Figures 7A,C,E, 8A,B; Table 1).

Finally, the *posterior* radial macrounit involves AHi in the periventricular stratum and PMCo in histologically indistinctly delimited, but molecularly distinguishable, intermediate, and superficial strata. This domain shows rostrolateral, rostromedial, and caudolateral molecular subunits (AHi, PMCoi, PMCos; Figures 7A,D, 8C–F; Table 1).

We also proposed tentatively in this model a novel fifth amygdalar pallial macrounit, called the *retroendopiriform* radial unit (*rep*). Its periventricular portion (REP nucleus) was the only part of this unit recognized previously (e.g., see REP in Paxinos and Franklin, 2007 mouse brain atlas). New molecular data and use of the amygdalar radial section plane suggested strongly its radial prolongation across an intermediate part (REPI; possibly equivalent to the classic ventral part of the basolateral nucleus, BLV) into the brain surface, where it’s REPCo specialization ends just rostral to the PLCo (REP; REPI; REPCo; Figures 7A,B; Table 1). The periventricular retroendopiriform nucleus also appears in other rodent brain atlases identified wrongly as a caudal part of either the dorsal or ventral endopiriform nuclei. Molecular data reveal such an association to be false since the respective profiles are very different (Garcia-Calero et al., 2020). On the other hand, it does not make sense to ascribe the radially disposed REP/REPI/REPCo complex to the adjacent amygdalo-piriform cortical area.

All these amygdalar radial units are located externally to the cortical pallium (Puelles et al., 2019; Garcia-Calero et al., 2020). This analysis identifies distinct periventricular, intermediate, and superficial component nuclei within each of the radial subunits, some of them previously unrecognized (Table 1). In general, regarding the resulting classification of pallial amygdalar nuclei, it was an objective of our study to depart as little as possible from conventional amygdalar terminology. Nevertheless, the novelties regarding particularly the previously poorly understood intermediate stratum turned out to make necessary some modified or novel terms. Those we propose are consistent with the studied gene patterns and the experimental radial glial observations that complemented this study (Garcia-Calero et al., 2020; Garcia-Calero and Puelles, submitted).

#### AHi, APir and CxA “Transition Areas” in the Context of the Radial Amygdalar Model

The classical concept of the amygdalar complex, transmitted essentially by specialized amygdala chapters in several treatises, describes traditionally several cortical transitional areas between some amygdalar corticoid elements and neighboring cortical structures such as hippocampal, entorhinal, or piriform cortex. This is the case for AHi and APir, both related topographically with the morphogenetically still *unexplained* amygdalar fissure (Krettek and Price, 1978; De Olmos et al., 1985, 2004; Alheid et al., 1995; Martínez-García et al., 2012; Olucha-Bordonau et al., 2015; Medina et al., 2017). The radial AHi/PMCo complex is located at the caudomedial region of the telencephalic temporal pole, protruding medialwards at the brain surface behind the medial amygdala (rostrally to the ventral end of the dentate gyrus and other parts of the hippocampus), and caudalwards with the prominent PMCo portion. According to our results, it is properly the *posterior* radial unit of the pallial amygdala (Garcia-Calero et al., 2020). What is identified as AHi in the literature is the periventricular stratum of this unit, which appears related radially to the intermediate and superficial PMCo corticoid mass, as was recognized already in some works (AHi, PMCo; Figures 8C–F; e.g., McDonald, 2003; Martínez-García et al., 2012; Olucha-Bordonau et al., 2015). We corroborated this radial relationship between AHi and PMCo by appropriate radial glia labeling experiments (Garcia-Calero et al., 2020). The *posterior* radial domain is rather large and shows subregions with peculiar molecular profiles. We accordingly subdivided it minimally into *rostrolateral*, *rostromedial*, and *caudolateral* radial subunits (Table 1). The rostromedial subunit it the one that lies caudal to the medial amygdala nuclei. The rostrolateral subunit ends superficially in a small separate superficial specialization lying medial to the PLCo, rather than at the PMCo.

AHi also shows in sagittal sections a clear-cut caudal molecular boundary with the ventral parasubiculum, subiculum, and hippocampus proper (AHi, Hi; De Olmos et al., 1985, 2004). Accordingly, we concluded that AHi is not a transitional area at all (consistently with its outside-in stratification vs. the hippocampal inside-out one). It is just an extensive *posterior* pallial amygdalar radial area (with three subdivisions), which neighbors the hippocampal formation and singularly lies largely *under* the caudal end of the lateral ventricle, rather than rostral to it, as the rest of the pallial amygdala.

We described the APir as a transitional zone between the ventral parts of the piriform and entorhinal cortical areas, placed next to the amygdala. Hence, we abandon its usual concept as a transitional area between the piriform cortex and the pallial amygdala (APir; Krettek and Price, 1978; De Olmos et al., 1985, 2004; Alheid et al., 1995; Martínez-García et al., 2012; Olucha-Bordonau et al., 2015; Medina et al., 2017). APir is a variant sort of olfactory allocortical structure (Garcia-Calero et al., 2020), which typically shows a wide superficial molecular layer receiving input from the lateral olfactory tract, and intermediate and deep layers (layer 1, layer 2, and layer 3; Figures 8G,H; De Olmos et al., 1985, 2004). Garcia-Calero et al. (2020) described in their Azin2-LacZ material subtle differences at the APir deep layer compared to the standard piriform cortex (see their Figure 5). We found as well that though APir lies at the piriform/entorhinal boundary, it only exists where this border zone adjoins the pallial amygdala (as if a patterning effect has spread partially from the amygdala into adjacent allocortex, but not into the whole width of the allocortex). The APir thus represents a special areal part of the olfactory outer allocortical ring (Puelles et al., 2019). This is the reason why we exclude it from the pallial amygdala proper. Tridimensionally, the APir is partly bent mediolaterally at the amygdalar fissure, like a flexed hand that holds in its concavity the caudal ends of the REP and BLP amygdalar formations. The REP radial unit labeled selectively in Azin2-LacZ material and also recognized selectively by other gene markers lies laterally to BLP, while APir appears lateral to REP (Garcia-Calero et al., 2020).

Another confusing aspect illuminated by the radial amygdala model is that in several literature sources the corticoid CxAC area belonging to the *basolateral* radial unit is misidentified as “APir.” This possibly also includes the correlative intermediate BLI component of the same radial unit (e.g., see Martínez-García et al., 2012; their Figures 6E, E’ 7D,E; where the BLP, the false “APir,” and the unlabeled subpial CxAC next to Pir form a clear-cut radial arrangement). This particular interpretation of APir is anti-intuitive. It poses a conceptual problem, since it implies the interposition of a full allocortical set of strata *within* the radial dimension of a nuclear amygdalar radial complex, rather than being parallel to the brain surface, as it theoretically should be. Such a cortical topological oddity never received specific attention, as far as we know, but the difficulty disappears with the redefinition of this putative “aberrant radial APir” subdomain as BLI, the caudal most intermediate part of the radial *BL*. Consistently with the overall concept of the *basal* radial unit, the superficial corticoid end of the basolateral subunit—CxAC— lies next to the caudal most piriform cortex and the retro-amygdalar *true* APir. The latter thus essentially lies caudally to the tri-dimensionally complex BLP/BLA/BLI/CxAC radial unit. Late-embryonic morphogenetic bending of the cortical wall at the amygdalar fissure causes the topologically caudal APir to be sectioned partially together with the end of the BLP/BLA/BLI/CxAC complex, which protrudes caudalwards.

As regards the conventional cortico-amygdalar (CxA) transitional area, also postulated to bridge the border between piriform cortex and pallial amygdala, we found this domain to be about double as long as it is usually represented in rodent atlases, with rostrocaudal differences leading to its subdivision into rostral and caudal portions (CxAR, CxAC; Figures 8A,B; Garcia-Calero et al., 2020). Both of them are strictly corticoid *amygdalar* structures, as shown by DiI labeling experiments on radial glia. These superficial corticoid parts belong respectively to the *lateral* and *basolateral* radial units. Thus, the classic CxA and its caudal continuation are integral superficial parts of the pallial amygdala.

## Checking the Pallial Amygdalar Radial Model: The Neurogenetic Pattern

### Neurogenetic Gradients Observed in Radial Units of the Pallial Amygdala

Neurogenesis in the pallial amygdala occurs in a straightforward *outside-in* pattern, which contrasts with the surrounding cortical pallial structures that uniformly show an *inside-out* birthdate pattern (Bayer, 1980a; McDonald, 2003; reviewed in Garcia-Calero et al., 2020). We examined previous tritiated-thymidine autoradiographic studies done mainly in rats (Bayer, 1980a,b, 1986; Bayer and Altman, 1987) and analogous mouse BrU experiments (Soma et al., 2009), together with relevant experiments shown in^1^. We reinterpreted these results within the amygdalar pallial radial model to re-assess neurogenetic timing according to the postulated amygdalar radial units.

We analyzed four of the main pallial amygdalar radial units (*lateral*, *basal*, *anterior*, and *posterior*) since the *retroendopiriform* radial unit did not appear in the published material. These four units clearly show an outside-in neurogenetic pattern, with superficial populations produced earlier than cells in the periventricular strata (Bayer, 1980a; McDonald, 2003; Garcia-Calero et al., 2020). The *anterior* radial unit completes its neurogenetic process earlier than the other radial units do. This unit shows a peak of neuron production at the ACo, the superficial layer, at E13 in the rat. BMA, the corresponding intermediate stratum, shows neurogenesis ranging from E13/14 to E16, with a peak at the E15 + E16 injection experiment (BMA, ACo; Figure 8A; Bayer, 1980a; Bayer and Altman, 1987^1^). Note the *anterior* radial unit has no postnatal periventricular stratum, because its derivatives accumulate in the mentioned two strata, or migrate away tangentially into surrounding subpallial areas, where similar early birthdates are observed; (Garcia-Calero et al., 2020; Garcia-Calero and Puelles, submitted). These results contrast with the neurogenetic timing at the periventricular strata of the *lateral* and *basolateral* radial units, which appears delayed to stages E17-E18 (L, BLP, and BMP; Figure 8B). As regards the *basomedial* subunit, we noted earliest neuron production at its superficial PLCo from E14 to E16, in contrast to labeled cells ranging from E15 to E18 at corresponding intermediate and deep structures. This pattern is similar to that at the neighboring periventricular L and BLP nuclei (Figures 8A,B; Bayer, 1980a; Bayer and Altman, 1987^1^).

In the *posterior* radial unit, the neurogenetic pattern detected at the intermediate and superficial strata, represented by corresponding parts of PMCo (PMCoi and PMCos, respectively), begins at the E13 + E14 labeling experiment at superficial levels (PMCos). There is a peak of superficial neurogenesis at E14 + E15 (PMCos; Figure 8C). The pattern continues in the intermediate stratum (PMCoi), labeled at E15 + E16 and E16 + E17 (PMCoi; Figure 8D; Bayer, 1980a^1^). On the other hand, the periventricular AHi nucleus of this unit shows a high number of labeled cells between stages E16 to E19, with a neurogenesis peak at E17 + E18 (AHi; Figures 8C–F; Bayer, 1980a^1^). This outside-in pattern of neurogenesis is similar (though slightly delayed) to the pattern of neuron production described in the other radial units in the pallial amygdala. These results confirm a similar outside-in neurogenesis pattern for all studied pallial radial amygdalar units, including the* posterior* one.

We also compared the neurogenetic pattern described in the *posterior* radial amygdalar unit, with the pattern observed in hippocampal subdivisions, present in the allocortical ring portion lying caudally adjacent to the *posterior* pallial amygdala. Indeed, the *posterior* radial amygdalar unit limits mainly with the CA1–CA3 hippocampal region, and only its caudolateral subdivision contacts with the subicular and possibly ventral entorhinal cortex (Garcia-Calero et al., 2020). All these hippocampal regions show an inside-out neurogenetic pattern, as described for other cortical structures (Bayer, 1980b; Bayer and Altman, 1987). In this complex, CA1 neurons are born earlier than CA3 neurons, with periventricular (alveus) cells in CA1 produced between E14 to E16, and layer 2 neurons born between E16–E20 (Bayer, 1980b; Bayer and Altman, 1987^1^). AHi periventricular cells are produced coincidently with layer 2 neurons in CA1. Also, a sandwich neurogenetic pattern has been reported in CA1-CA3, with pyramidal cells in layer 2 flanked superficially and deeply by older/earlier-born cells (Bayer, 1980b; Bayer and Altman, 1987). The latter may largely represent tangentially migrated subpallial interneurons.

Summarizing these results attending specifically to the neurogenetic pattern in the *periventricular* strata of the four studied radial units of the pallial amygdala, we observe neurogenetic periods ranging from E15/16 to E18/19 for the *lateral* and *basal* radial units and from E16/17 to E18/19 for the *posterior* radial unit. The *anterior* radial unit is represented periventricularly only by a glial palisade; however, the latest-born cells produced in this domain are located within BMA, and their neurogenesis ends at E15/16, indicating they are precocious relative to the intermediate counterparts in the other radial units, as happens with the superficial derivatives. We also detected earlier neurogenesis at the CxAR/CxAC than at the PLCo, which suggests a relatively earlier start of neurogenesis at the *lateral* and *basolateral* radial units, than at the *basomedial* radial unit. In this sense, we observe what seems a neurogenetic trend extending from the FMU unit closest to the subpallium (*anterior* radial unit) to the three FMUs that contact the allocortical ring (*lateral*, *basal*, and *posterior* radial units), implying possibly an overall amygdalar pallial gradient in this subpallio-pallial direction. However, it should be remembered that FMUs tend to control their proliferative and neurogenetic activities independently and heterochronically. It would be unexpected, though not impossible, that they regulate these patterns within a single gradient. More detailed comparative studies of neurogenesis than those we have now at our disposal might be able to detect discontinuities among the diverse amygdalar radial units.

### Neurogenesis in Allocortical APir

The radial amygdalar model contemplates the APir structure as a subtly modified areal part of the piriform cortex (Pir) residing at the outer border zone of the allocortical ring, next to the entorhinal cortex, but separate from the neighboring pallial amygdala (See Figure 1 in; Garcia-Calero et al., 2020). The entorhinal cortex separates APir from the ventral subiculum and other parts of hippocampal allocortex. APir is accordingly outside the amygdalar pallial complex but lies close in particular to the *retroendopiriform* and *basolateral* radial units. This concept contradicts previous amygdalar classifications that define APir as a transitional cortico-amygdalar domain (Krettek and Price, 1978; De Olmos et al., 1985, 2004; Alheid et al., 1995; Martínez-García et al., 2012; Olucha-Bordonau et al., 2015; Medina et al., 2017). We analyzed available data on the neurogenetic pattern in APir as well as neighboring areas of the allocortical ring (piriform cortex, hippocampus, and ERh). We reviewed tritiated-thymidine studies in rats and mice (Bayer, 1980a,b, 1986; Bayer and Altman, 1987; McDonald, 2003; Soma et al., 2009) and rat experiments published in^1^.

The earliest APir cells, located in layer 3 are produced mainly between E13 + E14 (injections at E13 + E14) to E14 + E15, but a few labeled cells were also observed in later experiments (APir layer 3; Figure 8G^1^). In contrast, the latest APir cells were produced mainly between E16 + E17 and E17 + E18, occupying layer 2, and forming cell clusters (layer 2, Figure 8H; injection E17 + E18^1^). These results indicate an inside-out pattern in APir, similar to the pattern described for other allocortical structures, but radically different from the neurogenetic pattern found at the pallial amygdala.

In the piriform cortex, an inside-out pattern was also described, with a caudal (earlier) to the rostral (later) neurogenetic pattern (Bayer, 1986). The earliest cells are produced at E14, and, in this sense, APir, representing a caudal areal subdomain within Pir, shows neurogenesis beginning at this earliest stage. It represents the starting locus of the olfactory cortex. In the rest of the allocortical ring (studied by Bayer and Altman, 1987), the entorhinal area shows also an inside-out pattern, with its earliest internal stratum cells born at E14, with a peak at E15. These data point to APir and the caudal piriform cortex as the oldest allocortical structures. From here, the olfactory cortex neurogenetic gradient points in the rostral direction towards the anterior piriform cortex. In contrast, the entorhinal cortex progresses from its earliest rostral starting point caudalwards (its lateral division develops earlier than its medial division) until reaching the hippocampal formation. Bayer and Altman emphasize in their interpretive schemata continuous gradients extending across several cortical areas. However, the theory of radial units with independent proliferation patterns suggests that the mirror orientation of birthday gradients observed across olfactory and entorhinal cortex domains probably reflect that these parts of the allocortical ring are independent FMUs, consistently with many other well-known differential histogenetic, structural, and functional aspects. We would expect a similar boundary between the entorhinal cortex and the hippocampus, with or without mirror aspects.

In summary, neurogenetic data consistently support the existence of an allocortical ring external to the pallial amygdala, which includes the APir as a singular olfactory subarea only present next to the amygdalar border of the allocortical ring. These cortical areas uniformly show an inside-out neurogenetic pattern that contrasts with the uniform amygdalar outside-in stratification pattern of timed birthdates. The results also point to APir and caudal Pir as the most precociously born structures in the allocortical ring. We may speculate whether this spatial and temporal correlation bespeaks of morphogenetic signals spreading into the cortex from the neighboring amygdalar field, which shows an even more precocious developmental start.

## Conclusion

In the present work, we show the importance of *radial morphogenetic studies* for clarifying current concepts about neuroanatomically complex brain regions and making possible future advances in our causal and comparative knowledge of central nervous system development and function. The formation of radial units begins *via* molecular regionalization and subsequent differential proliferation and neurogenesis leading to specific differential fates. This process is assisted by radial glial scaffolding, which builds a self-contained, probably molecularly diverse, skeleton for each one of the relatively independent *radial histogenetic units*, finally representing brain FMUs. We discussed in detail as an example of this type of study the recently proposed *radial model* *of the pallial amygdala* (Garcia-Calero et al., 2020). Compared to models based on topography in coronal sections or functionality, the new model has advantages, particularly towards aiding causal ontogenetic and evolutionary interpretation of amygdalar complexity.

Amygdalar models based in telencephalic development are helpful by showing pallial and subpallial macro-radial domains, divided later more or less hypothetically into pallial and subpallial subdivisions or sectors (Johnston, 1923; Holmgren, 1925; Smith-Fernandez et al., 1998; Puelles et al., 2000, 2016a, 2019; Puelles, 2013; Medina et al., 2004, 2017; Tole et al., 2005; García-López et al., 2008; Olucha-Bordonau et al., 2015). Objections to earlier amygdalar models result mainly from the recent realization that the much-used coronal section plane distorts amygdalar analysis, due to their obliquity relative to the observable amygdalar glial radial organization.

The present radial model of the pallial amygdala contemplates five main radial units, or macrounits, with a total of nine subdivisions, each displaying (with one exception) periventricular, intermediate and superficial strata, thus representing 27 distinct spatial amygdalar loci believed to have different neuronal populations, all of them differentiable neurogenetically from all bordering allocortical domains. We checked some of the assumptions of this model by reviewing the neurogenetic patterns described so far in the amygdalar region compared to neighboring allocortical areas, APir included. We corroborated a systematic outside-in pattern in four amygdalar radial units for which data were available, in contrast to the inverse inside-out pattern observed generally in all allocortex subdivisions. Among the studied four amygdalar radial units, the one with the most precocious neurogenesis (E13 in the rat) is the *anterior* radial unit.

## Author Contributions

EG-C designed this article. EG-C and LP wrote the article. All authors contributed to the article and approved the submitted version.

## Conflict of Interest

The authors declare that the research was conducted in the absence of any commercial or financial relationships that could be construed as a potential conflict of interest.

## Footnotes

^1^https://braindevelopmentmaps.org/
